# The expression of lncRNAs CASC2, NEAT1, LINC00299 in breast cancer tissues and their relationship with the XBP1 splicing rate in Iranian patients during 2014–2019: A cross‐sectional study

**DOI:** 10.1002/hsr2.1552

**Published:** 2023-09-11

**Authors:** Ghazal Orak, Hossein Babaahmadi Rezaei, Fereshteh Ameli, Fatemeh Maghsoodi, Maryam Cheraghzade, Maryam Adelipour

**Affiliations:** ^1^ Department of Clinical Biochemistry, School of Medicine Ahvaz Jundishapur University of Medical Sciences Ahvaz Iran; ^2^ Hyperlipidemia Research Center Ahvaz Jundishapur University of Medical Science Ahvaz Iran; ^3^ Department of Pathology, School of Medicine Tehran University of Medical Science Tehran Iran; ^4^ Department of Public Health Abadan University of Medical Sciences Abadan Iran; ^5^ Cellular and Molecular Research Center, Medical Basic Science Research Institute Ahvaz Jundishapur University of Medical Sciences Ahvaz Iran

**Keywords:** breast neoplasms, gene expression, long noncoding RNA, XBP1

## Abstract

**Background and Aims:**

Breast cancer is a leading cause of incidence and mortality in women globally. Identifying new molecular markers can aid in cancer diagnosis, targeted therapy, and treatment monitoring. This study aimed to measure the expression of the X‐box binding protein 1 (XBP1) gene, an index of the unfolded protein response (UPR), and long noncoding RNAs (lncRNAs), including Nuclear Enriched Abundant Transcript 1 (NEAT1), Cancer Susceptibility Candidate 2 (CASC2), and Long Intergenic Nonprotein Coding RNA 299 (LINC00299), as possible regulators of the UPR pathway.

**Methods:**

Total RNA was extracted from 40 samples of breast tumor tissues and their respective controls. The expression level of lncRNAs CASC2, NEAT1, and LINC00299 was quantified using reverse transcription‐polymerase chain reaction (RT‐PCR). The ratio of the spliced form of XBP1 to its unspliced form (XBP1u) was determined by PCR and electrophoresis.

**Results:**

The results showed a 2.8‐fold increase in the ratio of XBP1s/u in breast cancer tissues compared to adjacent nonmalignant samples (*p* < 0.05). Additionally, the level of lncRNAs NEAT1, CASC2, and LINC00299 in breast tumor tissues increased significantly by twofold, 1.5‐fold, and 2.3‐fold, respectively, compared to adjacent nonmalignant samples (*p* < 0.05).

**Conclusions:**

Based on the association between the expression of lncRNAs CASC2, LINC00299, and NEAT1 and the XBP1s/u ratio, these lncRNAs could be potential regulators of the UPR pathway. Also, CASC2 and NEAT1 genes could be suggested as suitable biomarkers to distinguish cancerous tissue from noncancerous breast tissue due to their significant increase in expression in cancerous samples compared to adjacent noncancerous.

## 1 INTRODUCTION

The incidence rate of breast cancer has increased and it is estimated that breast cancer has the highest incidence rate of cancer among females worldwide.^1^ Unfortunately, most of the time the disease is diagnosed in an advanced stage which is almost incurable and patients die in a short period. Many efforts have been made in recent years for the molecular identification of breast cancer to improve early diagnosis, targeted treatments, and treatment monitoring for patients.^2^

Paying attention to intracellular molecular pathways in cancer can reveal the pathways involved in cancer growth and progression, which can lead to targeted treatment and finding biomarkers for diagnosis, disease prognosis, and treatment monitoring. One such molecular pathway involved in cancer is endoplasmic reticulum stress (ERS). ERS conditions trigger the unfolded protein response (UPR), which can activate the inositol‐requiring enzyme 1 (IRE1) pathway. Activation of IRE1 causes the cleavage of a 26‐nucleotide intron from X‐box binding protein 1–unspliced (XBP1u) mRNA, which is subsequently converted into a transcription factor called XBP1s. The XBP1s transcription factor regulates a wide range of genes involved in endoplasmic reticulum homeostasis, cell viability, angiogenesis, metastasis, invasion, drug resistance, cell metabolism, and cell apoptosis by translocating to the nucleus.^3^

The UPR pathway can be regulated by long noncoding RNAs (lncRNAs), which are nonprotein‐coding RNAs that are more than 200 nucleotides in length and have a central role in different types of malignancies, including breast cancer.^4^, ^5^ LncRNAs and estrogen receptor stress work together in synergy to regulate the fate of tumor cells.^6^ Alterations in lncRNA expression can contribute to breast cancer metastasis by regulating cell invasion and migration.^7^ LncRNAs have been suggested as suitable biomarkers for cancer diagnosis and prognosis.^8^ Recently, noncoding RNAs have attracted the attention of researchers in the field of cancer because they can regulate gene expression by interacting with DNA, RNA, and proteins.^9^

Long Intergenic Nonprotein Coding RNA 299 (LINC00299) is a noncoding RNA that has been shown to increase the expression of XBP1 through its interaction with microRNA (miR‐135a‐5p).^10^ Recent studies have demonstrated that silencing LINC00299 through the LINC00299/miR‐490‐3p/Aurora Kinase A (AURKA) axis can significantly inhibit cell growth, increase apoptosis, and prevent the migration of vascular smooth muscle cells (VSMCs).^11^

Another lncRNA, Nuclear Enriched Abundant Transcript 1 (NEAT1), is upregulated in malignant tissues compared to nonmalignant tissues in several types of cancer, and therefore, it acts as an oncogene that can promote tumor cell progression. However, there is controversy about the oncogenic or tumor suppressor function of NEAT1 in cancer.^12^

Another lncRNA, Cancer Susceptibility Candidate 2 (CASC2), has been identified as a tumor suppressor in endometrial carcinoma and is known to control cell growth, migration, invasion, and cell death in several types of human cancers.^13^, ^14^ An increased expression level of CASC2 can induce cell apoptosis in vitro by arresting the cell cycle at the G1‐S checkpoint.^15^

Noncoding RNA Activating by DNA Damage (NORAD) plays a crucial role in protecting DNA and maintaining chromosomal stability.^4^ NORAD, recognized as an oncogene, has been linked to an unfavorable prognosis in different types of cancers.^16^ This biomarker is involved in several processes associated with carcinogenesis, including cell proliferation, apoptosis, invasion, and metastasis.^16^

In the present study, we aim to investigate the expression levels of NEAT1, LINC00299, and CASC2 genes in breast cancer samples compared to adjacent nonmalignant samples, as well as their relationship with the spliced XBP1 mRNA level. This investigation is crucial in determining the exact role of these genes in breast cancer.

## 2 MATERIALS AND METHODS

### 2.1 Patient selection and sample collection

In this cross‐sectional study, we collected 40 samples of breast cancer tumor tissues and their adjacent noncancerous tissues from the Cancer Institute at Imam Khomeini Hospital in Tehran, Iran. The average age of the patients studied was 52 years, with a median age of 52.5 years. Additionally, the average tumor size among the patients was 6.4 cm, with a median size of 4.0 cm. The sample size was determined based on a study by Yang et al.^17^ using the MedCalc statistical software with a power of 90% and α error of 5%. All participants were first‐time breast cancer patients who were undergoing surgery as their first treatment. Exclusion criteria included having a history of other diseases, taking medications, or receiving any kind of treatment. During surgery, one part of the malignant breast tissues and one part of their adjacent nonmalignant tissues were collected by the surgeon, frozen using liquid nitrogen, and stored at −80°C. The entire tissue that was removed during surgery was also transferred to a container containing 10% formalin and sent to the laboratory for histopathological studies. Table 1 shows the demographic information of the patients enrolled in the study. The Medical Ethics Committee of the Cancer Institute of Imam Khomeini Hospital and the Medical Ethics Committee of Jundishapur University of Medical Sciences approved this study (Ethics ID: IR. AJUMS. MEDICINE. REC.1400.068), and all volunteers signed informed consent forms. The study was conducted in accordance with the Declaration of Helsinki and clinical practice guidelines.

**Table 1:** Demographic characteristics of patients.

Parameters	Patients group *n*/d (%)
Age (years)	
<50	16 (40.0)
≥50	24 (60.0)
Race	
Persian	9 (22.5)
Azari	13 (32.5)
Gilaki and Mazani	4 (10.0)
Kurd	4 (10.0)
Lor	3 (7.5)
Unknown	7 (17.5)
Stage	
I–II	29 (72.5)
III	10 (25)
IV	1 (2.5)
Histology grade	
Grade I (low‐well differentiated)	4 (10.0)
Grade II (intermediate‐moderately differentiated)	22 (55.0)
Grade III (high‐poor differentiated)	14 (35.0)
Tumor size (cm)	
<5	25 (62.5)
≥5	15 (37.5)

### 2.2 RNA extraction from tissue samples and DNase treatment

First, the frozen tissues were homogenized using a mortar, pestle, and liquid nitrogen, and then total RNA was extracted from each sample using RiboExTM (Gene All) according to the manufacturer's protocol. The extracted RNA was kept at −70°C until used for cDNA synthesis. Electrophoresis was used to measure the quality of the extracted RNA, and a nanodrop spectrophotometer was used to check its quantity. In this study, DNase treatment was performed using DNase 1 kit (Sinaclon).

### 2.3 Synthesis of cDNA from tissue extracted‐RNA

In this study, cDNA was synthesized from RNA according to the manufacturer's protocol of the cDNA synthesis kit (Yekta Tehiz Azma).

### 2.4 Quantitative expression of NEAT1, CASC2, and LINC00299 genes

We measured the expression of NEAT1, CASC2, and LINC00299 genes with quantitative SYBR green master mix (Ampliqon) using Applied Biosystems QuantStudio3 system. Also, the gene expression of hypoxanthine phosphoribosyl transferase 1 was used as a reference gene. The sequences of the primers of the genes are listed in Table 2. The NORAD gene expression has been previously reported, in this study, the relationship between NORAD gene expression and XBP1 splicing was investigated.^18^

**Table 2:** Specifications of the primers used.

Genes	Forward primer (5′‐ > 3′)	Reverse primer (5′‐ > 3′)	Product length
LINC00299	TGCTGGGTTCGATGGTTCAA	TCCAGATAGGGCAGGTGACT	127
CASC2	TCAAAGAACCCTATTCCGAGT	TTGCACATCTCTCACTGAACA	110
NEAT1	AGTTTGAAAAGGCTAATCCAG	GACAACAGGCTAACTAACCC	146
HPRT	CCTGGCGTCGTGATTAGTG	TCAGTCCTGTCCATAATTAGTCC	125

Abbreviations: CASC2, Cancer Susceptibility Candidate 2; LINC00299, Long Intergenic Nonprotein Coding RNA 299; NEAT1, Nuclear Enriched Abundant Transcript 1.

#### 2.4.1 Real‐time **polymerase chain reaction** (QPCR)

For each qPCR reaction, 0.3 μL of each primer, 6.25 μL of SYBR Green, as well as 0.5 μL of cDNA were added to a final volume of 12 μL with distilled water. The reactions were performed in duplicate on the Applied Biosystems QuantStudio3. The PCR cycling conditions were set as follows: 15 min at 95°C for enzyme activation, followed by 40 cycles of 15 s at 95°C and 60 s at 60°C, with a linear heating step from 60 to 95°C to create a melting curve. The relative fold change of each gene in malignant samples compared to the respective control was calculated using the following formula:

$$\text{Delta}\;C_{t}=C_{t}\;\text{Gene}–C_{t}\;\text{HPRT}.$$

$$\text{Delta}\;\text{Delta}\;C_{t}=\Delta C_{t}\;\text{cancer}\;\text{tissue}–\Delta C_{t}\;\text{control}.$$

$$\text{Fold}\;\text{change}=2^{–(\Delta C_{t}\;\text{cancer}\;\text{tissue}–\Delta C_{t}\;\text{control})}.$$

The PCR efficiency for the genes was checked using LinReg PCR software. The results showed that the PCR efficiency values for all four genes fell between 1.9 and 2, indicating that the PCR reactions for these genes are efficient, as the values are within the desired range.

#### 2.4.2 Reverse Transcription‐PCR (RT‐PCR) and gel electrophoresis

RT‐PCR was used to determine the ratio of XBP1 Spliced/Unspliced in malignant and nonmalignant tissues. The PCR products were then analyzed by gel electrophoresis and the bands were visualized using GelDoc device. The intensity and area of each band were quantified using the ImageJ 2015 software.

### 2.5 Statistical analysis

Grouping was performed within each clinicopathological parameter, with patients divided into two or more groups based on each parameter. By using GraphPad Prism (9.5.1.733 version, Dotmatics company), first, normal distribution of the data was assessed using the Kolmogorov‐Smirnov test. As the data for all four genes did not follow a normal distribution, the Mann–Whitney and Kruskal–Wallis tests were used to compare two and multiple groups, respectively. The tests used in this study were two‐tailed. Additionally, receiver operating characteristic (ROC) curve analysis was conducted to assess the diagnostic value of each lncRNA, and the area under the ROC curve (AUC) was calculated to measure discriminatory capacity. The best sensitivity/specificity pair was selected based on the maximum likelihood ratio using GraphPad Prism. Furthermore, Spearman's correlation was employed to determine the relationship between genes. A *p* < 0.05 was considered significant. The relationship between gene expression and clinicopathological parameters was investigated using SPSS software (version 22, IBM company). For better comparison and interpretation of the data obtained from the electrophoresis of PCR products, the intensity and area of each band were converted into numerical quantities using ImageJ software (version 2022, NIH Image company).

## 3 RESULTS

In the histological examination, breast cancer was confirmed by the pathologist. Total RNA was isolated from freshly frozen tissues, and after cDNA synthesis, gene expression of NEAT1, CASC2, and LINC00299 was checked using quantitative RT‐PCR (QPCR).

### 3.1 The difference in CASC2, NEAT1, and LINC00299 expression in control and cancer groups

**Figure 1:**
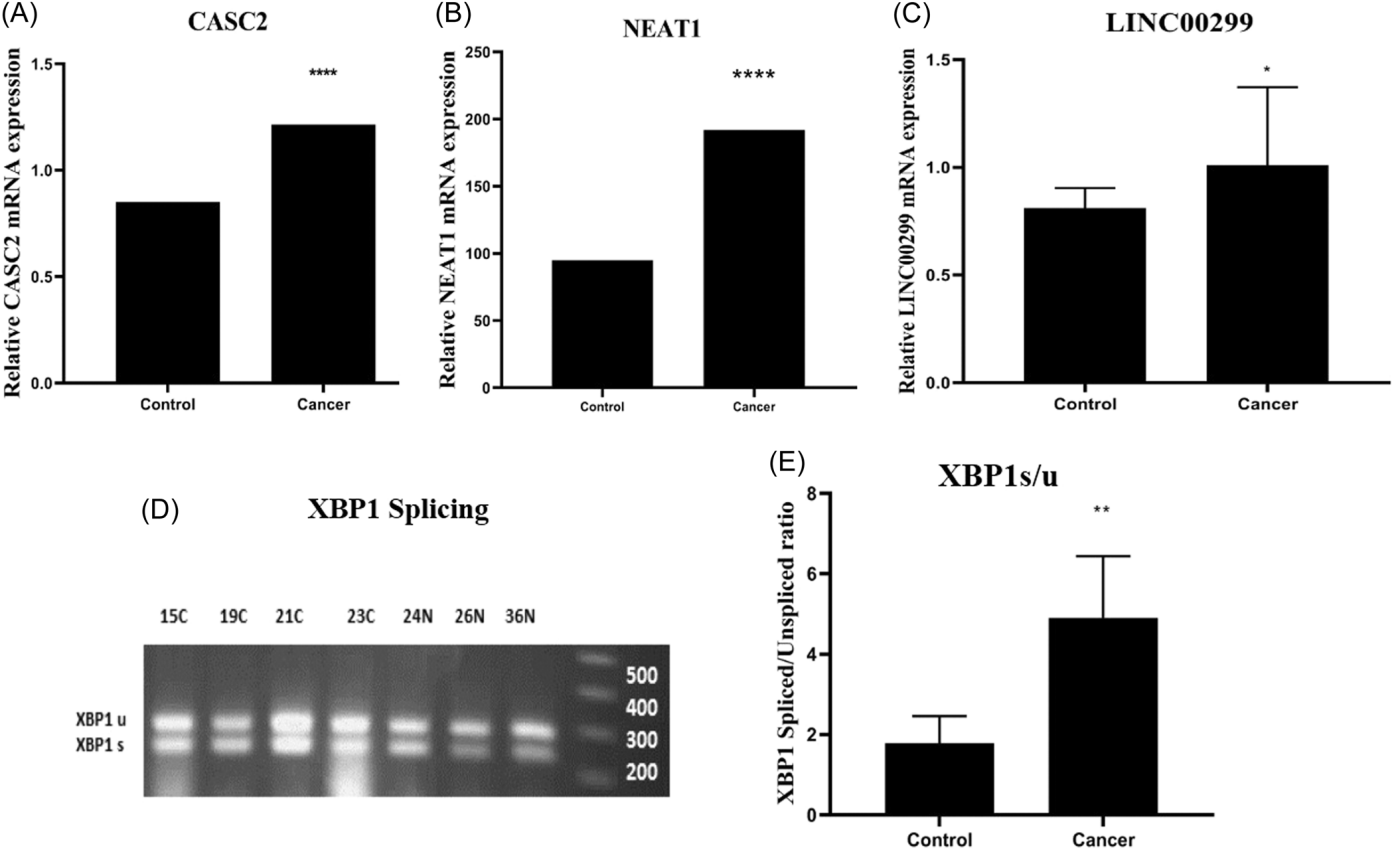
Comparison of Cancer Susceptibility Candidate 2 (CASC2), Nuclear Enriched Abundant Transcript 1 (NEAT1), Long Intergenic Nonprotein Coding RNA 299 (LINC00299), and X‐box binding protein 1 (XBP1) gene expression between control and cancer groups. (A) CASC2 gene expression in breast cancer tissues is significantly higher than noncancerous tissues. (B) NEAT1 gene expression in breast cancer tissues is significantly higher than noncancerous tissues. (C) LINC00299 gene expression in breast cancer tissues is significantly higher than noncancerous tissues. (D) Gel electrophoresis image showing the product of XBP1u (283 base pairs) and XBP1s (257 base pairs) obtained from polymerase chain reaction of cDNA samples of tumor and normal tissues (numbers on the top of the image indicate the number of the tissue, “C” is the symbol of cancer tissue, and “N” is the symbol of normal tissue). (E) Comparison of the relationship between the ratio of XBP1 Spliced/Unspliced in breast cancer compared to the adjacent normal tissue. The graphs are plotted based on the median with 95% confidence interval. Hypoxanthine phosphoribosyl transferase 1 was used as a reference gene (****p < 0.0001, **p < 0.0014, and *p < 0.03 compared to the nontumor group).

By using GraphPad Prism, data from QPCR indicated that the CASC2 gene expression was increased 1.5‐fold in the malignant breast tissues compared to their adjacent nonmalignant tissue (95% confidence interval [CI]: (−0.55) to (−0.17); *p* < 0.001, Figure 1A). Comparison of the level of lncRNA NEAT1 in malignant breast tissues with adjacent nonmalignant tissues showed a twofold increase of NEAT1 expression in breast cancer samples (95% CI: (−0.03) to (1.6); *p* < 0.001, Figure 1B). A 2.3‐fold increase was found in LINC00299 RNA in the malignant breast tissue compared to the adjacent nonmalignant tissue (95% CI: (0.00)–(0.48); *p* < 0.03, Figure 1C).

### 3.2 Association between expression of CASC2, NEAT1, and LINC00299 and clinicopathological parameters of breast cancer patients

By using SPSS software, evaluating the association between CASC2 gene expression and various clinicopathological features of patients showed a significant increase in CASC2 gene expression in tumors with smaller size, lower grade, lower stage, estrogen receptor positivity, and absence of lymphatic and vascular invasion (*p* < 0.05). However, there was no significant correlation between CASC2 expression and other clinicopathological features (*p* > 0.05, Table 3). Additionally, no significant relationship was found between NEAT1 RNA expression and any of clinicopathological features (*p* > 0.05, Table 3). In addition, the relationship between LINC00299 gene expression and various clinicopathological parameters of patients showed that there was a significant increase in LINC00299 gene expression in estrogen receptor‐positive tumors compared to estrogen receptor‐negative tumors (*p* < 0.05). No significant correlation was found between LINC00299 expression and other clinicopathological features (*p* > 0.05, Table 3).

**Table 3:** Correlation of CASC2, NEAT1, and LINC00299 expression with clinicopathological features of breast cancer.

Variable	CASC2		NEAT1		LINC00299	
	*p* Value	Maximum median	*p* Value	Maximum median	*p* Value	Maximum median
		Minimum		Minimum		Minimum
Tumor size (cm)						
<5	**0.001**	22.25	0.80	8.90	0.13	1.64
		0.21		0.80		0.09
		0.00		0.00		0.00
≥5		23.01		10.14		9.32
		0.07		0.80		0.02
		0.01		0.00		0.00
Grade						
I	**0.000**	12.55	0.38	7.12	0.67	1.00
		0.44		0.99		0.73
		0.04		0.09		0.00
II		23.02		10.14		9.32
		0.27		0.96		0.05
		0.01		0.00		0.00
III		22.25		8.90		1.33
		0.09		0.30		0.05
		0.00		0.00		0.00
Stage						
I–II	**0.002**	23.01	0.60	10.14	0.59	9.32
		0.21		0.99		0.07
		0.00		0.00		0.00
III		0.99		6.99		1.92
		0.06		0.19		0.05
		0.01		0.00		0.00
IV		0.11		1.36		0.02
		0.09		0.68		0.01
		0.07		0.01		0.00
ER						
Positive	**0.02**	22.25	0.57	10.14	**0.04**	9.32
		0.24		0.60		0.32
		0.00		0.00		0.00
Negative		1.08		8.90		1.64
		0.10		0.46		0.36
		0.01		0.00		0.00
PR						
Positive	0.23	1.08	0.07	7.12	.19	1.00
		0.20		0.49		0.32
		0.00		0.02		0.00
Negative		22.25		8.9		1.64
		0.10		0.26		0.03
		0.01		0.00		0.00
P53						
Positive	0.97	1.08	0.37	7.12	0.20	1.64
		0.29		0.53		0.06
		0.02		0.00		0.00
Negative		0.67		10.14		9.32
		0.30		3.01		0.45
		0.05		0.06		0.00
Necrosis						
Yes	0.12	12.55	0.39	10.14	0.20	9.32
		0.13		0.80		0.03
		0.00		0.00		0.00
No		23.01		7.12		1.92
		0.21		0.72		0.13
		0.02		0.00		0.00
Lymphatic invasion						
Yes	**0.01**	22.25	0.15	10.14	0.14	9.32
		0.11		0.39		0.03
		0.00		0.00		0.00
No		23.01		8.9		1.01
		0.26		0.99		0.11
		0.01		0.02		0.00
Vascular invasion						
Yes	**0.01**	22.25	0.15	10.14	0.36	9.32
		0.11		0.39		0.03
		0.00		0.00		0.00
No		23.01		8.9		1.01
		0.26		0.99		0.11
		0.01		0.02		0.00

*Note*: Bold numbers show the significant *p*‐value.

Abbreviations: CASC2, Cancer Susceptibility Candidate 2; ER, estrogen receptor; LINC00299, Long Intergenic Nonprotein Coding RNA 299; NEAT1, Nuclear Enriched Abundant Transcript 1; PR, progestrone receptor.

### 3.3 Sensitivity and specificity of the studied genes

By using GraphPad Prism, the specificity and sensitivity of the genes were determined by ROC curves (Table 4). We plotted ROC curves to determine quantification values of CASC2, NEAT1, and LINC00299 genes, in an attempt to differentiate cancer and noncancer groups patients. The cutoff levels were selected as the points on the ROC curves with the maximum sum of sensitivity and specificity using the Youden's index ([sensitivity + specificity] − 1).

**Table 4:** Sensitivity and specificity of the studied genes.

Genes	Sensitivity (%)	Specificity (%)	AUC	Cut‐off	95% confidence interval
CASC2	85	90	8.5	0.88	0.8004–0.9658
NEAT1	72	82	4.1	0.78	0.4735–0.7246
LINC00299	65	67	2.0	0.63	0.5035–0.7634

Abbreviations: AUC, area under the receiver operating characteristic curve; CASC2, Cancer Susceptibility Candidate 2; LINC00299, Long Intergenic Nonprotein Coding RNA 299; NEAT1, Nuclear Enriched Abundant Transcript 1.

### 3.4 The relationship between the XBP1 spliced/unspliced ratio in cancer and noncancer groups and its correlation with clinicopathological parameters of breast cancer patients

By using GraphPad Prism, the PCR data showed that the ratio of XBP1s/u in the malignant breast tissues was significantly increased by 2.8 times compared to the adjacent normal tissues (95% CI: (0.00)–(4.96); *p* < 0.001, Figure 1D,E). By using SPSS 22 software, the relationship between XBP1s/u ratio and different clinicopathological parameters of breast tissue is shown in Table 4. The data analysis showed that there was a significant increase in the XBP1 splicing rate in tumors with progesterone receptor‐positive and larger size compared to tumors with progesterone receptor‐negative and smaller size (*p* < 0.05). However, no significant correlation between XBP1s/u ratio and other clinicopathological parameters was found (*p* > 0.05, Table 5).

**Table 5:** Correlation between XBP1 spliced/unspliced ratio and clinicopathological parameters of breast cancer patients.

Variable	XBP1 spliced/unspliced	
	*p* Value	Maximum
		Median
		Minimum
Tumor size (cm)		
<5	**0.00**	11.21
		1.00
		0.00
≥5		16.21
		5.33
		0.00
Grade		
I	0.89	15.13
		1.00
		0.00
II		16.21
		1.00
		0.00
III		11.21
		1.00
		0.00
Stage		
I–II	0.93	15.13
		1.00
		0.00
III		16.21
		1.00
		0.00
IV		1.00
		1.00
		1.00
ER receptor		
Positive	0.29	15.13
		1.00
		0.00
Negative		9.42
		1.00
		0.00
PR receptor		
Positive	**0.03**	15.13
		1.00
		0.00
Negative		9.42
		1.00
		0.00
P53		
Positive	0.30	15.13
		0.75
		0.00
Negative		14.69
		4.23
		0.00
Necrosis		
Yes	0.33	16.21
		1.00
		0.00
No		15.13
		1.00
		0.00
Lymphatic invasion		
Yes	0.97	16.21
		1.00
		0.00
No		11.06
		1.00
		0.00
Vascular invasion		
Yes	0.97	16.21
		1.00
		0.00
No		11.06
		1.00
		0.00

*Note*: Bold numbers show the significant *p*‐value.

Abbreviations: ER, estrogen receptor; PR, progestrone receptor; XBP1, X‐box binding protein 1.

### 3.5 Relationship between XBP1 splicing rate and expression of NORAD, CASC2, NEAT1, and LINC00299 genes in breast cancer tissues

By using GraphPad Prism, the relationship between XBP1s/u ratio and the expression of NORAD, CASC2, NEAT1, and LINC00299 genes in breast cancer patients is shown in Table 5. In the previous study, NORAD gene expression was investigated in cancerous tissues and adjacent nonmalignant tissue, and the results showed that NORAD gene expression in cancerous tissues is about five times higher than adjacent nonmalignant tissues.^18^ In this study, the relationship between NORAD gene expression and XBP1 splicing rate was measured. Spearman's test indicated that there was a significant negative correlation between XBP1s/u ratio and NORAD (*r* = −0.3964; 95% CI: (−0.57) to (−0.18); *p* = 0.001), NEAT1 (*r* = −0.1938; 95% CI: (−0.54) to (−0.15); *p* = 0.001), and LINC00299 (*r* = −0.2324; 95% CI: (−0.43) to (−0.006); *p* = 0.03) gene expression. Also, there is a significant positive correlation between XBP1 S/U ratio and CASC2 (*r* = 0.4016; 95% CI: (−0.19) to (−0.57); *p* = 0.001) gene expression.

## 4 DISCUSSION

The results of the current study showed an increased expression of CASC2 in breast malignant tissues compared to the adjacent nonmalignant tissue (*p* < 0.001, Figure 1A). Evaluation of the relationship between the level of lncRNA CASC2 and different clinicopathological parameters of breast cancer tissues showed that in tumors with smaller size, lower grade, lower stage, estrogen receptor positive, and no lymphatic and vascular invasion, the expression of CASC2 gene was significantly higher (*p* < 0.05, Table 3). These results suggest a possible tumor suppressor role of lncRNA CASC2. In this regard, Peng Li and his colleagues investigated the effect of CASC2 on the proliferation of gastric cancer cells in 2016, and their results confirmed the increased expression of CASC2 in GC cells by Q‐PCR method (*p* < 0.05).^19^ Their results showed that CASC2 effectively inhibits cell proliferation by modulating the G1‐S checkpoint and inducing cell apoptosis, indicating that CASC2 lncRNA may have a tumor suppressor role.^19^

In addition, our findings showed that the expression of lncRNA NEAT1 was significantly increased in malignant breast tissues compared to the adjacent nonmalignant tissues (*p* < 0.001, Figure 1B). However, no significant correlation was found between increased NEAT1 expression in breast cancer tissues and clinicopathological parameters of patients (*p* > 0.05, Table 3). In this regard, Huang et al. reported the higher expression of lncRNA NEAT1 in pancreatic cancer tissues compared to the respective noncancerous tissues.^20^ Their data showed that NEAT1 silencing suppresses cell proliferation through induction of cell cycle arrest and increased apoptosis in pancreatic cancer cells, suggesting an oncogenic role of this lncRNA in pancreatic cancer as NEAT1 can be a new diagnostic and therapeutic target for pancreatic cancer.^20^ However, a study by Masashi Idogawa et al. demonstrated that NEAT1 is a direct transcriptional target of p53, and suppression of NEAT1 by p53 led to a reduction in the inhibitory effect of p53 on cancer cell proliferation.^21^ Therefore, their results show that p53 and NEAT1 contribute to various biological functions such as tumor suppression by forming a transcriptional network.^21^ The difference in the type of cancerous samples and laboratory conditions may be the reason for the different action of NEAT in various studies.

LINC00299 gene, as a lncRNA, has a 2.3‐fold increase in expression in malignant breast tissue compared to the adjacent normal tissue (*p* < 0.03, Figure 1C). Evaluation of the relationship between LINC00299 gene expression and various clinicopathological features of patients showed a significantly higher expression of the LINC00299 gene in estrogen receptor‐positive tumors compared to estrogen receptor‐negative tumors (*p* < 0.05, Table 3). Yong Liu et al. showed that during atherosclerosis, proliferation of endothelial cells and vascular smooth muscle cells was associated with increased expression of Linc00299 and decreased expression of miR‐490‐3p in atherosclerosis patients. Their findings indicated that knockdown of Linc00299 and overexpression of miR‐490‐3p led to suppression of cell proliferation, induction of apoptosis, and inhibition of VSMC migration.^11^ Their study revealed the central effect of the Linc00299/miR‐490‐3p/AURKA axis on the regulation of cell proliferation and migration in atherosclerosis.^11^

In addition, the ratio of XBP1s/u in malignant breast tissue was found to be significantly increased by 2.8‐fold compared to adjacent nonmalignant tissue (*p* < 0.001, Figure 1E). Further analysis revealed a significant correlation between XBP1 splicing rate and various clinicopathological features of patients, including positive progesterone receptor status and larger tumor size (*p* < 0.05, Table 5). Moreover, we observed a negative correlation between the XBP1s/u ratio and the Delta Ct values of lncRNAs NORAD, NEAT1, and LINC00299, representing their gene expression levels in cancer tissues. Considering that a lower *C*_t_ value indicates higher gene expression, it suggests that an increase in gene expression of these lncRNAs is associated with an increase in the splicing rate of XBP1. Conversely, the gene expression of CASC2 showed a positive correlation, indicating that an increase in CASC2 gene expression is associated with a decrease in the splicing rate of XBP1. These results suggest a regulatory role of these lncRNAs in the UPR pathway. Our findings are in line with those of Davies et al., who reported that a higher XBP1s/u ratio was associated with poor survival, absence of estrogen and progesterone receptors, and grade 3 tumors in breast cancer patients undergoing endocrine therapy in the United States.^22^ However, this study had some limitations, such as the lack of access to overall survival or disease‐free survival data of patients and breast Carcinoma In Situ tissues.

## 5 CONCLUSION

This study demonstrated that the expression levels of lncRNAs NEAT1, CASC2, and LINC00299 in malignant breast tissue were significantly increased by twofold, 1.5‐fold, and 2.3‐fold, respectively (*p* < 0.05) compared to adjacent noncancerous tissues. Additionally, the XBP1s/u ratio in breast cancer tissue was increased by approximately 2.8‐fold compared to the adjacent normal tissue (*p* < 0.05). Data analysis revealed a positive correlation between the expression of XBP1s/u ratio and NORAD, NEAT1, and LINC00299 gene expression, while an increase in CASC2 gene expression was associated with a decrease in XBP1 splicing. Therefore, it appears that these lncRNAs negatively regulate the UPR pathway. However, further studies are required to determine the exact functions of these lncRNAs in breast cancer initiation and development. Furthermore, based on the ROC curve results, CASC2 and NEAT1 genes may be suitable biomarkers for breast cancer diagnosis.

## AUTHOR CONTRIBUTIONS

**Ghazal Orak**: Conceptualization; data curation; investigation; methodology; project administration; software; writing—original draft; writing—review and editing. **Hossein Babaahmadi rezaei**: Data curation; investigation; writing—review and editing. **Fereshteh Ameli**: Methodology; writing—review and editing. **Fatemeh Maghsoodi**: Formal analysis; software; writing—review and editing. **Maryam Cheraghzade**: Investigation; writing—review and editing. **Maryam Adelipour**: Conceptualization; data curation; investigation; methodology; supervision; writing—review and editing.

## CONFLICT OF INTEREST STATEMENT

The authors declare no conflict of interest.

## ETHICS STATEMENT

This study was approved by the Medical Ethics Committee of the Cancer Institute of Imam Khomeini Hospital and also by the Medical Ethics Committee of Jundishapur University of Medical Sciences (Ethics ID: IR. AJUMS. MEDICINE. REC.1400.068). All volunteers signed informed consent forms. The study was conducted in accordance with the Declaration of Helsinki and clinical practice guidelines.

## TRANSPARENCY STATEMENT

The lead author Maryam Adelipour affirms that this manuscript is an honest, accurate, and transparent account of the study being reported; that no important aspects of the study have been omitted; and that any discrepancies from the study as planned (and, if relevant, registered) have been explained.

## Data Availability

The data that support the findings of this study are available from the corresponding author upon a reasonable request.
